# Supplementary material for: The learnability of Pauli noise
Source: Nat Commun. 2023 Jan 4;14:52. doi: 10.1038/s41467-022-35759-4 (PMC9813261; doi:10.1038/s41467-022-35759-4)
Supplement: Supplementary file 1 — Supplementary Information [file 41467_2022_35759_MOESM1_ESM.pdf]

Supplementary Information: The learnability of Pauli noise

Senrui Chen,[1,*] Yunchao Liu,[2,*] Matthew Otten,[3] Alireza Seif,[1] Bill Fefferman,[4] and Liang Jiang[1,*]

[1]*Pritzker School of Molecular Engineering, University of Chicago, IL 60637, USA*
[2]*Department of Electrical Engineering and Computer Sciences, University of California, Berkeley, CA 94720, USA*
[3]*HRL Laboratories, LLC, 3011 Malibu Canyon Rd., Malibu, CA 90265, USA*
[4]*Department of Computer Science, University of Chicago, IL 60637, USA*

(Dated: December 8, 2022)

CONTENTS

S1. Preliminaries S1

S2. Theory on the learnability of Pauli noise S2
- A. Assumptions and definitions S2
- B. Learnability of individual Pauli fidelity S3
- C. Characterization of learnable space via algebraic graph theory S7
- D. Learnability under no-crosstalk assumption S13
- E. Learnability of Pauli error rates S14

S3. Additional details about the numerical simulations S15

S4. Justification for the claim in Sec. S2 C S16

Supplementary References S19

S1. PRELIMINARIES

Define P^n to be the n-qubit Pauli group modulo its center. We can label any Pauli operator $P_a \in \mathsf{P}^n$ with a $2n$-bit string a. Specifically, we define $P_{\mathbf{0}}$ to be the identity operator I. We will use the notations P_a and a interchangeably when there is no confusion.

The *pattern* of an n-qubit Pauli operator P_a, denoted as $\mathrm{pt}(P_a)$, is an n-bit string that takes 0 at the jth bit if P_a equals to I at the jth qubit and takes 1 otherwise. For example, $\mathrm{pt}(XYIZI) = \mathrm{pt}(XXIXI) = 11010$.

An n-qubit *Pauli diagonal map* Λ is a linear map of the following form

$$\Lambda(\cdot) = \sum_{a\in\mathsf{P}^n} p_a P_a(\cdot)P_a, \tag{1}$$

where $\boldsymbol{p} \coloneqq \{p_a\}_a$ are called the *Pauli error rates*. If Λ is further a CPTP map, which corresponds to the condition $p_a \geq 0$ and $\sum_a p_a = 1$, then it is called a *Pauli channel*. An important property of Pauli diagonal maps is that their eigen-operators are exactly the 4^n Pauli operators. Thus, an alternative expression for Λ is

$$\Lambda(\cdot) = \frac{1}{2^n}\sum_{b\in\mathsf{P}^n} \lambda_b \operatorname{Tr}(P_b(\cdot))P_b, \tag{2}$$

* S.C. and Y.L. contributed equally to this work (alphabetical order). Correspondence and requests for materials should be addressed to S.C. (csenrui@uchicago.edu), Y.L. (yunchaoliu@berkeley.edu) or L.J. (liang.jiang@uchicago.edu).

where $\boldsymbol{\lambda} \coloneqq \{\lambda_b\}_b$ are called the *Pauli fidelities* or *Pauli eigenvalues* [1–3]. These two sets of parameters, $\boldsymbol{p}$ and $\boldsymbol{\lambda}$, are related by the Walsh-Hadamard transform

$$\lambda_b = \sum_{a\in\mathsf{P}^n} p_a(-1)^{\langle a,b\rangle}, \quad p_a = \frac{1}{4^n}\sum_{b\in\mathsf{P}^n} \lambda_b(-1)^{\langle a,b\rangle}, \tag{3}$$

where $\langle a, b\rangle$ equals to 0 if P_a, P_b commute and equals to 1 otherwise.

For a general linear map $\mathcal{E}$, define its *Pauli twirl* as

$$\mathcal{E}^P \coloneqq \sum_{a\in\mathsf{P}^n} \mathcal{P}_a\mathcal{E}\mathcal{P}_a. \tag{4}$$

Here we use the calligraphic $\mathcal{P}_a$ to represent the unitary channel of Pauli gate P_a, $\mathcal{P}_a(\cdot) \coloneqq P_a(\cdot)P_a$. The Pauli twirl of any linear map (quantum channel) is a Pauli diagonal map (Pauli channel). When we talk about the Pauli fidelities of a non-Pauli channel, we are effectively referring to the Pauli fidelities of its Pauli twirl.

S2. THEORY ON THE LEARNABILITY OF PAULI NOISE

In this section, we give a precise characterization of what information in the Pauli noise channel associated with Clifford gates can be learned in the presence of state-preparation-and-measurement (SPAM) noise. Our results show that certain Pauli fidelities of a noisy multi-qubit Clifford gate cannot be learned in a SPAM-robust manner, even with the assumption that single-qubit gates can be perfectly implemented. The proof is related to the notion of *gauge freedom* in the literature of gate set tomography [4]. We note that the results presented in this section emphasizes on the no-go part, *i.e.*, some information about the Pauli noise is (SPAM-robustly) unlearnable even with many favorable assumptions on the experimental conditions. As shown in the main text, the learnable information about Pauli noise can be extracted in a much more practical setting using cycle benchmarking [5] and its variant.

A. Assumptions and definitions

We focus on an n-qubit quantum system. Below are our assumptions on the noise model.

- **Assumption 1.** All single qubit unitary operation can be perfectly implemented.
- **Assumption 2.** A set of multi-qubit Clifford gates $\mathfrak{G} \coloneqq \{\mathcal{G}\}$ can be implemented and are subject to gate-dependent Pauli noise, *i.e.*, $\widetilde{\mathcal{G}} = \mathcal{G}\circ\Lambda_{\mathcal{G}}$ where $\Lambda_{\mathcal{G}}$ is some n-qubit Pauli channel.
- **Assumption 3.** Any state preparation and measurement can be implemented, up to some fixed Pauli noise channel $\mathcal{E}^S$ and $\mathcal{E}^M$, respectively.
- **Assumption 4.** The Pauli noise channels appearing in the above assumptions satisfy that all Pauli fidelities and Pauli error rates are strictly positive.

Assumption 1 is motivated by the fact that the noise of single-qubit gates are usually much smaller than that of multi-qubit gates on today's hardware. Such approximation is widely adopted in the literature [5, 6] with slight modifications. In Assumption 2, we view every Clifford gate as an n-qubit gate, and allow the noise to be n-qubit. This means we are taking all crosstalk into account.

A Clifford gate acting on a different (ordered) subset of qubits is viewed as a different gate and can thus have a different noise channel (*e.g.*, CNOT_{12}, CNOT_{21}, CNOT_{23} have different noise channels.) We will discuss the no-crosstalk situation in Sec. S2 D. The rationale for assuming Pauli noise in Assumption 2 and 3 is that we can always use randomized compiling [6, 7] to tailor general noise into Pauli channels. Finally, Assumption 4 is mostly for technical convenience. The requirement of positive Pauli error rates roughly implies the Pauli channels are at the interior of the CPTP polytope, and will be useful later in constructing valid gauge transformations. The requirement of positive Pauli fidelities is also reasonable for any physically interesting noise model.

Specifying a Clifford gate set $\mathfrak{G}$, a *noise model* satisfying our assumptions is determined by the Pauli channels describing gate noise and SPAM noise. We can thus view a noise model as a collection of Pauli fidelities, denoted as $\mathcal{N} = \{\mathcal{E}^S, \mathcal{E}^M, \Lambda\}$, where $\mathcal{E}^{S/M} = \{\lambda_a^{S/M}\}_a$ describes the SPAM noise and $\Lambda = \{\lambda_a^{\mathcal{G}}\}_{a,\mathcal{G}}$ describes the gate noise. We note that this is an example of *parametrized gate set* in the language of gate set tomography [4].

In order to gain information about an unknown noise model, one needs to conduct *experiments*. In the circuit model, any experiment can be described by some state preparation, a sequence of quantum gates, and some POVM measurements. An experiment conducted with different underlying noise model would yield different measurement outcome distributions. Explicitly, consider an (ideal) experiment with initial state ρ_0, gate sequence $\mathcal{C}$, POVM measurements $\{E_o\}_o$. Denote the noisy implementation of these objects within a certain noise model $\mathcal{N}$ with a tilde. Then the experiment effectively maps $\mathcal{N}$ to a probability distribution $p_{\mathcal{N}}(o) = \mathrm{Tr}(\widetilde{E}_o(\widetilde{\mathcal{C}}(\widetilde{\rho}_0)))$. We call two noise models $\mathcal{N}_1$, $\mathcal{N}_2$ *indistinguishable* if for all possible experiments we have $p_{\mathcal{N}_1} = p_{\mathcal{N}_2}$, and distinguishable otherwise.

Definition 1 (Learnable and unlearnable function)**.** *A function f of noise models is learnable if*

$$f(\mathcal{N}_1) \neq f(\mathcal{N}_2) \implies \mathcal{N}_1, \mathcal{N}_2 \text{ are distinguishable}, \tag{5}$$

for any noise models $\mathcal{N}_1$, $\mathcal{N}_2$. In contrast, f is unlearnable if there exist indistinguishable noise models $\mathcal{N}_1$, $\mathcal{N}_2$ such that $f(\mathcal{N}_1) \neq f(\mathcal{N}_2)$.

Note that the above definition of "learnable" does not necessarily mean that the value of the function can be learned. However, throughout this paper whenever some function is "learnable" according to Definition 1, it is also learnable in the stronger sense that we can design an experiment to estimate it up to arbitrarily small error with high success probability.

In the language of gate set tomography, an unlearnable function is a *gauge-dependent* quantity of the gate set [4]. On the other hand, any learnable function can in principle be learned to arbitrary precision. In the following, we will focus the learnability of the functions of the gate noise, including individual and multiplicative combinations of Pauli fidelities.

B. Learnability of individual Pauli fidelity

We first study the learnability of individual Pauli fidelities associated with a Clifford gate. This has been an open problem in recent study of quantum benchmarking. Perhaps surprisingly, we obtain the following simple criteria on the learnability of Pauli fidelities with any Clifford gate.

Theorem 1. *With Assumptions 1-4, for any n-qubit Clifford gate $\mathcal{G}$ and Pauli operator P_a, the Pauli fidelity $\lambda_a^{\mathcal{G}}$ is unlearnable if and only if $\mathcal{G}$ changes the pattern of P_a, i.e.,* $\mathrm{pt}(\mathcal{G}(P_a)) \neq \mathrm{pt}(P_a)$.

The fact that certain Pauli fidelities are SPAM-robustly unlearnable is observed in some recent works [5, 7–9], described as "degeneracy" of the noise model. Our work is the first to give a rigorous argument for this by establishing connections to gate set tomography. As an example, for the CNOT and SWAP gates, we can immediately list its learnable and unlearnable Pauli fidelities in Table S1. We note that, the no-go theorem holds even under the no-crosstalk assumption as will be discussed in Sec. S2 D, so introducing ancillary qubits or other multi-qubit Clifford gates cannot help resolve the unlearnability.

Gate	Learnable	Unlearnable
CNOT	$\lambda_{II}, \lambda_{ZI}, \lambda_{IX}, \lambda_{ZX}, \lambda_{XZ}, \lambda_{YY}, \lambda_{XY}, \lambda_{YZ}$	$\lambda_{IZ}, \lambda_{XI}, \lambda_{ZZ}, \lambda_{XX}, \lambda_{IY}, \lambda_{YI}, \lambda_{ZY}, \lambda_{YX}$
SWAP	$\lambda_{II}, \lambda_{XX}, \lambda_{XY}, \lambda_{XZ}, \lambda_{YX}, \lambda_{YY}, \lambda_{YZ}, \lambda_{ZX}, \lambda_{ZY}, \lambda_{ZZ}$	$\lambda_{IX}, \lambda_{IY}, \lambda_{IZ}, \lambda_{XI}, \lambda_{YI}, \lambda_{ZI}$

Supplementary Table S1. Learnability of individual Pauli fidelity of CNOT and SWAP.

Before going into the proof, we make several remarks about Theorem 1. The correct interpretation of the no-go result in Theorem 1 is that certain Pauli fidelities cannot be learned in a fully SPAM-robust manner. If one has some pre-knowledge that the SPAM noise is much weaker than the gate noise, there exist methods to give a pretty good estimate of those unlearnable Pauli fidelities, according to physical constraints. See the discussions in the main text. On the other hand, it is observed that the product of certain unlearnable Pauli fidelities can be learned in a SPAM-robust manner, such as $\lambda_{XI} \cdot \lambda_{XX}$ for the CNOT gate [5]. We will characterize the learnability of this kind of products of Pauli fidelities in the next subsection.

Proof of Theorem 1. We start with the "only if" part, which is equivalent to saying that $\mathrm{pt}(P_a) = \mathrm{pt}(\mathcal{G}(P_a))$ implies $\lambda_a^{\mathcal{G}}$ being learnable. The condition $\mathrm{pt}(\mathcal{G}(P_a)) = \mathrm{pt}(P_a)$ implies $\mathcal{G}(P_a)$ is equivalent to P_a up to some local unitary transformation, *i.e.*, there exists a product of single-qubit unitary gates $\mathcal{U} := \bigotimes_{j=1}^{n} \mathcal{U}_j$ such that

$$\mathcal{U} \circ \mathcal{G}(P_a) = P_a. \tag{6}$$

Now we design the following experiments parameterized by a positive integer m,

- Initial state: $\rho_0 = (I + P_a)/2^n$,
- POVM measurement: $E_{\pm 1} = (I \pm P_a)/2$,
- Circuit: $\mathcal{C}^m = (\mathcal{U} \circ \mathcal{G})^m$.

Consider the measurement probability by running these experiments within a noise model $\mathcal{N}$.

$$\begin{aligned} p_{\pm 1}^{(m)}(\mathcal{N}) &= \mathrm{Tr}\left(\widetilde{E}_{\pm 1} \widetilde{\mathcal{C}}^m(\widetilde{\rho}_0)\right) \\ &= \mathrm{Tr}\left(\frac{I \pm P_a}{2} \cdot \left(\mathcal{E}^M \circ (\mathcal{U} \circ \mathcal{G})^m \circ \mathcal{E}^S\right)\left(\frac{I + P_a}{2^n}\right)\right) \\ &= \mathrm{Tr}\left(\frac{I \pm P_a}{2} \cdot \frac{I + \lambda_a^M \left(\lambda_a^{\mathcal{G}}\right)^m \lambda_a^S P_a}{2^n}\right) \\ &= \frac{1 \pm \lambda_a^M \left(\lambda_a^{\mathcal{G}}\right)^m \lambda_a^S}{2}. \end{aligned} \tag{7}$$

Recall that $\lambda_a^{S/M}$ is the Pauli fidelity of the SPAM noise channel for P_a. The expectation value is

$$\mathbb{E}^{(m)}(\mathcal{N}) = \lambda_a^M \left(\lambda_a^{\mathcal{G}}\right)^m \lambda_a^S. \tag{8}$$

If we take the ratio of expectation values of two experiments with consecutive m, we obtain (recall that all these Pauli fidelities are strictly positive by Assumption 4)

$$\mathbb{E}^{m+1}(\mathcal{N})/\mathbb{E}^{m}(\mathcal{N}) = \lambda_a^{\mathcal{G}}. \tag{9}$$

This implies that if two noise model assign different values for $\lambda_a^{\mathcal{G}}$, the above experiments would be able to distinguish between them. By definition 1, we conclude $\lambda_a^{\mathcal{G}}$ is learnable.

Next we prove the "if" part. Fix an n-qubit Clifford gate $\mathcal{G}$. Let P_a be any Pauli operator such that $\mathrm{pt}(\mathcal{G}(P_a)) \neq \mathrm{pt}(P_a)$. We will show that $\lambda_a^{\mathcal{G}}$ is unlearnable by explicitly constructing indistinguishable noise models that assign different values to $\lambda_a^{\mathcal{G}}$.

Recall that any experiment involves a noisy initial state $\tilde{\rho}_0$, a noisy measurement $\{\widetilde{E}_l\}_l$, and a quantum circuit consisting of noiseless single-qubit gates $\mathcal{U} \coloneqq \bigotimes_{j=1}^n \mathcal{U}_j$ and noisy multi-qubit Clifford gates $\widetilde{\mathcal{T}}$. Now, introduce an invertible linear map $\mathcal{M} : \mathcal{L}(\mathcal{H}_{2^n}) \to \mathcal{L}(\mathcal{H}_{2^n})$, and consider the following transformation

$$\begin{aligned}
&\widetilde{\rho}_0 \mapsto \mathcal{M}(\widetilde{\rho}_0), \quad \widetilde{E}_l \mapsto (\mathcal{M}^{-1})^\dagger(\widetilde{E}_l),\\
&\bigotimes_{j=1}^n \mathcal{U}_j \mapsto \mathcal{M} \circ \bigotimes_{j=1}^n \mathcal{U}_j \circ \mathcal{M}^{-1},\\
&\widetilde{\mathcal{T}} \mapsto \mathcal{M} \circ \widetilde{\mathcal{T}} \circ \mathcal{M}^{-1}.
\end{aligned} \tag{10}$$

One can immediately see that any measurement outcome distribution $p_l \coloneqq \mathrm{Tr}(\widetilde{E}_l \widetilde{\mathcal{C}}(\widetilde{\rho}_0))$ remains unchanged via such transformation. Therefore the noise models related by this transformation are indistinguishable. This is called a *gauge transformation* in the literature of gate set tomography [4]. To use this idea for the proof, we start with a noise model $\mathcal{N}$ and construct a map $\mathcal{M}$ such that

1. The transformation yields a physical noise model $\mathcal{N}'$ satisfying Assumptions 1-5 in Sec. S2 A.

2. The two noise models $\mathcal{N}$, $\mathcal{N}'$ assign different values to $\lambda_a^{\mathcal{G}}$.

Starting with a generic noise model $\mathcal{N} = \{\mathcal{E}^S, \mathcal{E}^M, \Lambda\}$ satisfying the assumptions, we construct the gauge transform map $\mathcal{M}$ as follows. Since $\mathrm{pt}(\mathcal{G}(P_a)) \neq \mathrm{pt}(P_a)$, there exists an index $i \in [k]$ such that one and only one of $(P_a)_i$ and $\mathcal{G}(P_a)_i$ equals to I. Let $\mathcal{M}$ be the single-qubit depolarizing channel on the i-th qubit,

$$\mathcal{M} \coloneqq \mathcal{D}_i \otimes \mathcal{I}_{[n]\backslash i}, \tag{11}$$

where the single-qubit depolarizing channel is defined as

$$\forall P \in \{I, X, Y, Z\}, \quad \mathcal{D}(P) = \begin{cases} P, & \text{if } P = I, \\ \eta P, & \text{otherwise,} \end{cases} \tag{12}$$

for some parameter $0 < \eta < 1$. We will specify the value of η later.

Now we calculate the transformed noise model $\mathcal{N}' = \{\mathcal{E}^{S'}, \mathcal{E}^{M'}, \Lambda'\}$. The SPAM noise channels are transformed as

$$\mathcal{E}^{S'} = \mathcal{M}\mathcal{E}^S, \quad \mathcal{E}^{M'} = \mathcal{E}^M \mathcal{M}^{-1}, \tag{13}$$

both of which are still Pauli diagnoal maps. Thanks to our Assumption 4, as long as η is sufficiently close to 1, they can be shown to be Pauli channels.

Next, the single-qubit unitary gates are transformed as

$$\mathcal{M}\left(\bigotimes_{j=1}^{n}\mathcal{U}_j\right)\mathcal{M}^{-1}=\mathcal{D}_i\mathcal{U}_i\mathcal{D}_i^{\dagger}\otimes\bigotimes_{j\neq i}\mathcal{U}_j=\bigotimes_{j}\mathcal{U}_j, \tag{14}$$

since the single-qubit deplorizing channel commutes with any single-qubit unitary. This implies the single-qubit unitary gates are still noiseless.

Finally, consider an arbitrary n-qubit Clifford gate $\mathcal{T}$. We show that the transformed noisy gate takes the form $\widetilde{\mathcal{T}}'=\widetilde{\mathcal{T}}\circ\Lambda'_{\mathcal{T}}$ where $\Lambda'_{\mathcal{T}}$ is still a Pauli channel, with the Pauli fidelities updated as follows.

$$\lambda_b^{\mathcal{T}\prime}=\begin{cases}\eta\lambda_b^{\mathcal{T}}, & \text{if } \mathrm{pt}(P_b)_i=0 \text{ and } \mathrm{pt}(\mathcal{T}(P_b))_i=1,\\ \eta^{-1}\lambda_b^{\mathcal{T}}, & \text{if } \mathrm{pt}(P_b)_i=1 \text{ and } \mathrm{pt}(\mathcal{T}(P_b))_i=0,\\ \lambda_b^{\mathcal{T}}, & \text{if } \mathrm{pt}(P_b)_i=\mathrm{pt}(\mathcal{T}(P_b))_i.\end{cases} \tag{15}$$

We give a proof for the first case. Note that

$$\begin{aligned}\mathcal{M}\circ\widetilde{\mathcal{T}}\circ\mathcal{M}^{-1}&=\mathcal{D}_i\circ\widetilde{\mathcal{T}}\circ\mathcal{D}_i^{-1}\\&=\mathcal{D}_i\circ\mathcal{T}\circ\Lambda_{\mathcal{T}}\circ\mathcal{D}_i^{-1}\\&=\mathcal{T}\circ(\mathcal{T}^{-1}\circ\mathcal{D}_i\circ\mathcal{T}\circ\Lambda_{\mathcal{T}}\circ\mathcal{D}_i^{-1})\\&=:\mathcal{T}\circ\Lambda'_{\mathcal{T}},\end{aligned} \tag{16}$$

where we use $\mathcal{D}_i$ as a shorthand for $\mathcal{D}_i\otimes\mathcal{I}_{[n]\backslash i}$. The transformed noise channel can be written as

$$\Lambda'_{\mathcal{T}}=\mathcal{T}^{-1}\circ\mathcal{D}_i\circ\mathcal{T}\circ\Lambda_{\mathcal{T}}\circ\mathcal{D}_i^{-1}. \tag{17}$$

Let us calculate its action on arbitrary P_b.

$$\begin{aligned}\Lambda'_{\mathcal{T}}(P_b)&=(\mathcal{T}^{-1}\circ\mathcal{D}_i\circ\mathcal{T}\circ\Lambda_{\mathcal{T}}\circ\mathcal{D}_i^{-1})(P_b)\\&=(\eta^{-1})^{\mathrm{pt}(P_b)_i}(\mathcal{T}^{-1}\circ\mathcal{D}_i\circ\mathcal{T}\circ\Lambda_{\mathcal{T}})(P_b)\\&=\lambda_b^{\mathcal{T}}(\eta^{-1})^{\mathrm{pt}(P_b)_i}(\mathcal{T}^{-1}\circ\mathcal{D}_i\circ\mathcal{T})(P_b)\\&=\eta^{\mathrm{pt}(\mathcal{T}(P_b))_i}\lambda_b^{\mathcal{T}}(\eta^{-1})^{\mathrm{pt}(P_b)_i}\;P_b.\end{aligned} \tag{18}$$

Thus, $\Lambda'_{\mathcal{T}}$ is indeed a Pauli diagonal map with Pauli fidelities given by Eq. (15). The fact that $\Lambda'_{\mathcal{T}}$ is guaranteed to be a CPTP map by choosing appropriate η will be verified later. Specifically, if we take $\mathcal{T}$ to be the Clifford gate $\mathcal{G}$ that we are interested in, we have $\lambda_a^{\mathcal{G}\prime}=\eta\lambda_a^{\mathcal{G}}$ or $\lambda_a^{\mathcal{G}\prime}=\eta^{-1}\lambda_a^{\mathcal{G}}$. In either case, $\lambda_a^{\mathcal{G}\prime}\neq\lambda_a^{\mathcal{G}}$. This means the two indistinguishable noise model $\mathcal{N}$, $\mathcal{N}'$ indeed assign different values to $\lambda_a^{\mathcal{G}}$.

We now verify that $\mathcal{N}'$ is indeed a physical noise model and satisfies Assumptions 1-4. We have already shown that single-qubit unitary gates remain noiseless and that all gate noise and SPAM noise are described by Pauli diagonal maps. The only thing left is to make sure all these Pauli diagonal maps are CPTP and satisfy the positivity constraints in Assumption 4. According to Eq. (13) and (15), any Pauli fidelity λ_b of either SPAM noise or gate noise is transformed to one of the following $\lambda'_b\in\{\lambda_b,\eta\lambda_b,\eta^{-1}\lambda_b\}$, so $\lambda_b>0$ implies $\lambda'_b>0$. On the other hand, any transformed Pauli error rate can be bounded by

$$\begin{aligned}p'_c&=\frac{1}{4^n}\sum_{b\in\mathsf{P}^n}(-1)^{\langle b,c\rangle}\lambda'_b\\&\geq\frac{1}{4^n}\sum_{b\in\mathsf{P}^n}\left((-1)^{\langle b,c\rangle}\lambda_b-(\eta^{-1}-1)\lambda_b\right)\\&\geq p_c-(\eta^{-1}-1).\end{aligned} \tag{19}$$

To ensure every $p'_c > 0$, we can choose $1 > \eta > (p_{\min} + 1)^{-1}$ with $p_{\min}$ being the minimum Pauli error rate among all Pauli channels of both SPAM and gate noise, which is possible since $p_{\min} > 0$ by Assumption 4. This means each transformed Pauli diagonal maps are completely positive (CP). To see they are also trace-preserving (TP), just notice from Eq. (13), (15) that $\lambda'_{\mathbf{0}} = \lambda_{\mathbf{0}} = 1$ always holds. Now we conclude that $\mathcal{N}'$ is indeed a physical noise model satisfying all the assumptions. Combining with the reasoning in the last paragraph, we see $\lambda_a^{\mathcal{G}}$ is unlearnable. This completes our proof. □

C. Characterization of learnable space via algebraic graph theory

We have characterized the learnability of individual Pauli fidelities associated with any Clifford gates in Theorem 1. Here, we want to understand the learnablity for a general function of the gate noise. We first show that, in our setting, any measurement outcome probability in experiment can be expressed as a polynomial of Pauli fidelities of gate and SPAM noise, and each term in the polynomial can be learned via a CB experiment (see Sec. S4 for details). Therefore, it suffices to study the monomials, *i.e.*, products of Pauli fidelities. For each Pauli fidelity $\lambda_a^{\mathcal{G}}$, we define the *logarithmic Pauli fidelity* as $l_a^{\mathcal{G}} := \log \lambda_a^{\mathcal{G}}$ ($\lambda_a^{\mathcal{G}} > 0$ by Assumption 4). It then suffices to study the learnability of linear functions of the logarithmic Pauli fidelities. An alternative reason to only study this class of function is that, under a weak noise assumption, we have $l_a \to 0$, so we can express any function of the noise model as a linear function of l_a under a first order approximation. Note that similar approaches have been explored in the literature [10, 11].

Since we are working with Assumption 1-4 which takes all crosstalk into account, we treat the noise channel for each gate in $\mathfrak{G}$ as n-qubit. The number of independent Pauli fidelities we are interested in is thus

$$|\Lambda| = |\mathfrak{G}| \cdot 4^n. \tag{20}$$

Denote the space of all (real-valued) linear function of logarithmic Pauli fidelities as F, then we have $F \cong \mathbb{R}^{|\Lambda|}$. A function $f \in F$ uniquely corresponds to a vector $\boldsymbol{v} \in \mathbb{R}^{|\Lambda|}$ by $f(\boldsymbol{l}) = \boldsymbol{v} \cdot \boldsymbol{l} = \sum_{a,\mathcal{G}} v_{a,\mathcal{G}} l_a^{\mathcal{G}}$. We will use the vector to refer to the linear function when there is no ambiguity.

Denote the set of all learnable function in F as F_L (in the sense of Def. 1). As shown in the following lemma, F_L forms a linear subspace in F, so we call F_L the *learnable space*.

Lemma 1. *F_L is a linear subspace of F.*

Proof. Given $\boldsymbol{v}_1, \boldsymbol{v}_2 \in F_L$, consider the learnability of $\boldsymbol{v}_1 + \boldsymbol{v}_2$. For any noise models $\mathcal{N}_1$, $\mathcal{N}_2$,

$$\begin{aligned} (\boldsymbol{v}_1 + \boldsymbol{v}_2) \cdot \boldsymbol{l}_{\mathcal{N}_1} \neq (\boldsymbol{v}_1 + \boldsymbol{v}_2) \cdot \boldsymbol{l}_{\mathcal{N}_2} &\implies \boldsymbol{v}_1 \cdot \boldsymbol{l}_{\mathcal{N}_1} \neq \boldsymbol{v}_1 \cdot \boldsymbol{l}_{\mathcal{N}_2} \text{ or } \boldsymbol{v}_2 \cdot \boldsymbol{l}_{\mathcal{N}_1} \neq \boldsymbol{v}_2 \cdot \boldsymbol{l}_{\mathcal{N}_2} \\ &\implies \mathcal{N}_1, \mathcal{N}_2 \text{ are distinguishable.} \end{aligned} \tag{21}$$

Thus $\boldsymbol{v}_1 + \boldsymbol{v}_2 \in F_L$. We also have $\boldsymbol{v} \in L \implies k\boldsymbol{v} \in F_L$ for all $k \in \mathbb{R}$. Therefore, F_L forms a vector space in $\mathbb{R}^{|\Lambda|}$. □

Our goal is to give a precise characterization of the learnable space F_L. For example, we may want to know the dimension of F_L, which represents the learnable degrees of freedom for the noise. This is also the maximum number of linearly-independent equations about the logarithmic Pauli fidelities we can expect to extract from experiments. Conversely, the unlearnable degrees of freedom roughly correspond to the number of independent gauge transformations. We summarize these definitions as follows.

Definition 2. *Given a Clifford gate set* $\mathfrak{G}$*, the learnable degrees of freedom* $\mathrm{LDF}(\mathfrak{G})$ *and unlearnable degrees of freedom* $\mathrm{UDF}(\mathfrak{G})$ *are defined as, respectively,*

$$\mathrm{LDF}(\mathfrak{G}) \coloneqq \dim(F_L), \quad \mathrm{UDF}(\mathfrak{G}) \coloneqq |\Lambda| - \dim(F_L). \tag{22}$$

Our approach is to relate F_L to certain properties of a graph defined as follows.

Definition 3 (Pattern transfer graph)**.** *The pattern transfer graph associated with a Clifford gate set* $\mathfrak{G}$ *is a directed graph* $G = (V, E)$ *constructed as follows:*

- $V(G) = \{0,1\}^n$.
- $E(G) = \{e_{a,\mathcal{G}} \coloneqq (\mathrm{pt}(P_a),\ \mathrm{pt}(\mathcal{G}(P_a)) \mid \forall\ P_a \in \mathsf{P}^n,\ \mathcal{G} \in \mathfrak{G}\}$.

The 2^n vertices each corresponds to a possible Pauli pattern. The $|E| = |\Lambda| = |\mathfrak{G}| \cdot 4^n$ edges each corresponds to a Pauli operator and a Clifford gate, describing how the Clifford gate evolves the pattern of the Pauli operator. One can also think each edge corresponds to a unique Pauli fidelity ($e_{a,\mathcal{G}} \leftrightarrow \lambda_a^{\mathcal{G}}$). The rationale for only tracking the Pauli pattern is that we assume the ability to implement noiseless single-qubit unitaries, which makes the actual single-qubit Pauli operators unimportant. Fig. 2 of main text shows the pattern transfer graphs for a CNOT gate, a SWAP gate, and a gate set of CNOT and SWAP, respectively.

Next, we give some definitions from graph theory (see [12, 13]). A *chain* is an alternating sequences of vertices and edges $z = (v_0, e_1, v_1, e_2, v_2, ..., v_{q-1}, e_q, v_q)$ such that each edge satisfies $e_k = (v_{k-1}, v_k)$ or $e_k = (v_k, v_{k-1})$. A chain is *simple* if it does not contain the same edge twice. A closed chain (*i.e.*, $v_0 = v_q$) is called a *cycle*. If an edge e_k in a chain satisfies $e_k = (v_{k-1}, v_k)$, it is called an *oriented edge*. A chain consists solely of oriented edges is called a *path*. A closed path is called a *oriented cycle* or a *circuit*. A graph is called *strongly connected* if there is a path from every vertex to every other vertex. A graph is called *weakly connected* if there is a chain from every vertex to every other vertex. The number of (strongly or weakly) *connected components* is the minimum number of partitions of the vertex set $V = V_1 \cup \cdots \cup V_c$ such that each subgraph generated by a vertex partition is (strongly or weakly) connected.

We can equip a graph with vector spaces. Following the notations of [13, Sec. II.3], the *edge space* $C_1(G)$ of a directed graph G is the vector space of all linear functions from the edges $E(G)$ to $\mathbb{R}$. By construction, $C_1(G) \cong \mathbb{R}^{|\Lambda|} \cong F$. Every linear function of the logarithmic Pauli fidelities naturally corresponds to a linear function of the edges according to the label of the edges ($l_a^{\mathcal{G}} \leftrightarrow e_{a,\mathcal{G}}$). Again, we use vectors in $\mathbb{R}^{|\Lambda|}$ to refer to elements of $C_1(G)$. The inner product on $C_1(G)$ is defined as the standard inner product on $\mathbb{R}^{|\Lambda|}$.

There are two subspaces of $C_1(G)$ that is of special interest. For a simple cycle z in G, we assign a vector $\boldsymbol{v}_z \in C_1(G)$ as follows

$$\boldsymbol{v}_z(e) = \begin{cases} +1, & e \in z,\ e \text{ is oriented.} \\ -1, & e \in z,\ e \text{ is not oriented.} \\ 0, & e \notin z. \end{cases} \tag{23}$$

The *cycle space* $Z(G)$ is the linear subspace of $C_1(G)$ spanned by all cycles $\boldsymbol{v}_z$ in G.

Given a partition of vertices $V = V_1 \cup V_2$ such that there is at least one edge between V_1 and V_2, a *cut* is the set of all edges $e = (u, v)$ such that one of u, v belongs to V_1 and the other belongs to V_2. For each cut p we assign an vector $\boldsymbol{v}_p \in C_1(G)$ as follows

$$\boldsymbol{v}_p(e) = \begin{cases} +1, & e \in p,\ e \text{ goes from } V_1 \text{ to } V_2. \\ -1, & e \in p,\ e \text{ goes from } V_2 \text{ to } V_1. \\ 0, & e \notin p. \end{cases} \tag{24}$$

The *cut space* $U(G)$ is the linear subspace of $C_1(G)$ spanned by all cuts $\boldsymbol{v}_p$ in G. Note that different partition of vertices may result in the same cut vector if G is unconnected.

Lemma 2. *[13, Sec. II.3, Theorem 1] The edge space $C_1(G)$ is the orthogonal direct sum of the cycle space $Z(G)$ and the cut space $U(G)$, whose dimensions are given by*

$$\dim(Z(G)) = |E| - |V| + c(G), \quad \dim(U(G)) = |V| - c(G), \tag{25}$$

where $c(G)$ is the number of weakly connected components of G.

In some cases, we are more interested in circuits (oriented cycles) instead of general cycles. The following lemma gives a sufficient condition when the cycle spaces have a circuit basis, *i.e.* the cycle space is spanned by oriented cycles.

Lemma 3. *[12, Theorem 7] A directed graph has a circuit basis if it is strongly connected, or it is a union of strongly connected subgraphs.*

With all the graph theoretical tools introduced above, we are ready to present the main result of this section.

Theorem 2. *Under the Assumptions 1-4. For any $\mathfrak{G}$, $F_L \cong Z(G)$. Explicitly, a linear function $f_{\boldsymbol{v}}(\boldsymbol{l}) = \boldsymbol{v} \cdot \boldsymbol{l}$ is learnable if and only if $\boldsymbol{v}$ belongs to the cycle space $Z(G)$.*

We give the proof at the end of this section. The proof involves two parts. The first is to show that every cycle is learnable using a variant of cycle benchmarking [5], thus the cycle space belongs to the learnable space. The second part is to show that every cut induces a gauge transformation [4], and thus the learnable space must be orthogonal to the cut space, which implies it lies in the cycle space.

We remark that Theorem 1 can be viewed as a corollary of Theorem 2. This is because an individual Pauli fidelity $\lambda_a^{\mathcal{G}}$ whose Pauli pattern changes (*i.e.*, $\mathrm{pt}(P_a) \neq \mathrm{pt}(\mathcal{G}(P_a))$) corresponds to an simple edge in the pattern transfer graph, which does not belong to the cycle space and is thus unlearnable. On the other hand, a Pauli fidelity without Pauli pattern change corresponds to a self-loop in the pattern transfer graph, which belongs to the cycle space by definition, and is thus learnable.

Combing Theorem 2 with Lemma 2 leads to the following.

Corollary 3. *The learnable and unlearnable degrees of freedom associated with $\mathfrak{G}$ are given by*

$$\mathrm{LDF}(\mathfrak{G}) = |\mathfrak{G}| \cdot 4^n - 2^n + c(\mathfrak{G}), \quad \mathrm{UDF}(\mathfrak{G}) = 2^n - c(\mathfrak{G}), \tag{26}$$

where $c(\mathfrak{G})$ is the number of connected components of the pattern transfer graph associated with $\mathfrak{G}$.

Note that the unlearnable degrees of freedom always constitute an exponentially small portion, though they can grow exponentially.

Examples of some gate sets are given in Table S2 and Figure S1. One can notice some interesting properties. The UDF of CNOT and SWAP equals to 2 and 1, respectively, but a gate set containing both has UDF = 2. This means UDF is not "additive". The interdependence between different gates can give us more learnable degrees of freedom. However, Corollary 3 implies that the UDF of a gate set cannot be smaller than the UDF of any of its subset. This is because adding new gates can only decrease the number of connected components $c(\mathfrak{G})$ of the pattern transfer graph.

Number of qubits n	Gate set $\mathfrak{G}$	Number of parameters $\lvert\Lambda\rvert = 4^n \lvert\mathfrak{G}\rvert$	UDF($\mathfrak{G}$)
2	CNOT	16	2
2	SWAP	16	1
2	{CNOT, SWAP}	32	2
3	$\{\mathrm{CNOT}_{12}, \mathrm{CNOT}_{23}, \mathrm{CNOT}_{31}\}$	192	6
3	CIRC_3	64	4

Supplementary Table S2. The unlearnable degrees of freedom of some gate sets. Here CIRC_3 is the circular permutation on 3 qubits. UDF is calculated by applying Corollary 3 to the corresponding pattern transfer graph in Fig. 2 of main text and Fig. S1.

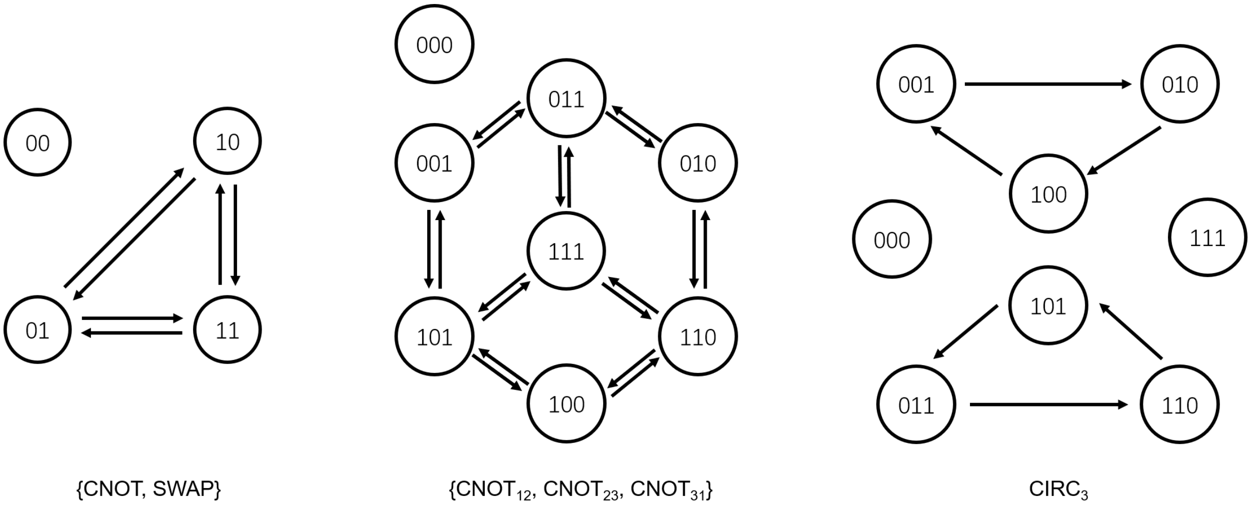

Supplementary Figure S1. Pattern transfer graphs for {CNOT, SWAP}, $\{\mathrm{CNOT}_{12}, \mathrm{CNOT}_{23}, \mathrm{CNOT}_{31}\}$, and CIRC_3. For clarity, we omit labels of the edges, multiple edges, and self-loop. These omissions do not change the cut space of the graph.

Proof of Theorem 2. The proof is divided into showing $Z(G) \subseteq F_L$ and $F_L \subseteq Z(G)$ (up to the natural isometry between F and $C_1(G)$).

$Z(G) \subseteq F_L$: Roughly, this is equivalent to saying that all cycles are learnable. We will first show that the pattern transfer graph always has a circuit basis, and then show that the linear function associated with each circuit can be learned using a variant of cycle benchmarking protocol [5].

We begin by showing that the pattern transfer graph G associated with a gate set $\mathfrak{G}$ is a union of strongly connected subgraphs. This is equivalent to saying that for any vertices $u, v \in V(G)$, if there is a path from u to v, there must be a path from v to u. It suffices to show that for each edge $e = (u, v)$ there is a path from v to u, since any path is just concatenation of edges. By definition, the existence of $e = (u, v)$ implies there exists $P \in \mathsf{P}^n$ and $\mathcal{G} \in \mathfrak{G}$ such that $\mathrm{pt}(P) = u$ and $\mathrm{pt}(Q) = v$ where $Q \coloneqq \mathcal{G}(P)$. Since a Clifford gate is a permutation on the Pauli group, there must exist some integer $d > 0$ such that $\mathcal{G}^d = \mathcal{I}$, thus $P = \mathcal{G}^{d-1}(Q)$, which induces the following path from v to u:

$$(\mathrm{pt}(Q),\ e_{Q,\mathcal{G}},\ \mathrm{pt}(\mathcal{G}(Q)),\ e_{\mathcal{G}(Q),\mathcal{G}},\ \mathrm{pt}(\mathcal{G}^2(Q)),\ \cdots,\ \mathrm{pt}(\mathcal{G}^{d-2}(Q)),\ e_{\mathcal{G}^{d-1}(Q),\mathcal{G}},\ \mathrm{pt}(\mathcal{G}^{d-1}(Q))).$$

One can verify this is a path according to the definition of G. This shows that G is indeed a union of strongly connected subgraphs. According to Lemma 3, G has a circuit basis that spans the cycle space $Z(G)$.

Now we show that every circuit in G represents a learnable function. Consider an arbitrary circuit $z = (v_0, e_1, v_1, e_2, v_2, ..., v_{q-1}, e_q, v_q \equiv v_0)$. For each $k = 1...q$, the edge e_k corresponds to a

Pauli operator $P_k \in \mathsf{P}^n$ and a Clifford gate $\mathcal{G}_k \in \mathfrak{G}$ such that $\mathrm{pt}(P_k) = v_{k-1}$ and $\mathrm{pt}(Q_k) = v_k$ where $Q_k := \mathcal{G}_k(P_k)$. On the other hand, since $\mathrm{pt}(Q_k) = \mathrm{pt}(P_{k+1})$, there exists a product of single qubit unitaries $\mathcal{U}_k$ such that $P_{k+1} = \mathcal{U}_k(Q_k)$ for $k = 1...q$ (where we define $P_{q+1} := P_1$, as $\mathrm{pt}(Q_q) = \mathrm{pt}(P_1)$ by assumptions). Consider the following gate sequence,

$$\mathcal{C} := \mathcal{U}_q\mathcal{G}_q\mathcal{U}_{q-1}\mathcal{G}_{q-1}\cdots\mathcal{U}_1\mathcal{G}_1 \tag{27}$$

One can see that $\mathcal{C}(P_1) = P_1$. Now we design the following experiments parameterized by a positive integer m,

- Initial state: $\rho_0 = (I + P_1)/2^n$,
- POVM measurement: $E_{\pm 1} = (I \pm P_1)/2$,
- Circuit: $\mathcal{C}^m = (\mathcal{U}_q\mathcal{G}_q\mathcal{U}_{q-1}\mathcal{G}_{q-1}\cdots\mathcal{U}_1\mathcal{G}_1)^m$.

Consider the outcome distribution generated by running these experiments within a noise model $\mathcal{N}$.

$$\begin{aligned} p_{\pm 1}^{(m)}(\mathcal{N}) &= \mathrm{Tr}\left(\widetilde{E}_{\pm 1}\widetilde{\mathcal{C}}^m(\widetilde{\rho}_0)\right) \\ &= \mathrm{Tr}\left(\frac{I \pm P_1}{2}\cdot\left(\mathcal{E}^M \circ \left(\mathcal{U}_q\widetilde{\mathcal{G}}_q\cdots\mathcal{U}_1\widetilde{\mathcal{G}}_1\right)^m \circ \mathcal{E}^S\right)\left(\frac{I + P_1}{2^n}\right)\right) \\ &= \mathrm{Tr}\left(\frac{I \pm P_1}{2}\cdot\frac{I + \lambda_{P_1}^M\left(\lambda_{P_q}^{\mathcal{G}_q}\cdots\lambda_{P_2}^{\mathcal{G}_2}\lambda_{P_1}^{\mathcal{G}_1}\right)^m\lambda_{P_1}^S P_1}{2^n}\right) \\ &= \frac{1 \pm \lambda_{P_1}^M\left(\lambda_{P_q}^{\mathcal{G}_q}\cdots\lambda_{P_2}^{\mathcal{G}_2}\lambda_{P_1}^{\mathcal{G}_1}\right)^m\lambda_{P_1}^S}{2}. \end{aligned} \tag{28}$$

The expectation value is

$$\mathbb{E}^{(m)}(\mathcal{N}) = \lambda_{P_1}^M\left(\lambda_{P_q}^{\mathcal{G}_q}\cdots\lambda_{P_2}^{\mathcal{G}_2}\lambda_{P_1}^{\mathcal{G}_1}\right)^m\lambda_{P_1}^S. \tag{29}$$

If we take the ratio of expectation values of two experiments with consecutive m, we obtain (recall that all these Pauli fidelities are strictly positive by Assumption 4)

$$\mathbb{E}^{m+1}(\mathcal{N})/\mathbb{E}^m(\mathcal{N}) = \lambda_{P_q}^{\mathcal{G}_q}\cdots\lambda_{P_2}^{\mathcal{G}_2}\lambda_{P_1}^{\mathcal{G}_1}. \tag{30}$$

This implies that if two noise models have different values for the product of Pauli fidelities $\lambda_{P_q}^{\mathcal{G}_q}\cdots\lambda_{P_2}^{\mathcal{G}_2}\lambda_{P_1}^{\mathcal{G}_1}$, the above experiments would be able to distinguish between them. Therefore, $\lambda_{P_q}^{\mathcal{G}_q}\cdots\lambda_{P_2}^{\mathcal{G}_2}\lambda_{P_1}^{\mathcal{G}_1}$ is a learnable function. By taking the logarithm of this expression, we see that $f(\boldsymbol{l}) := \sum_{k=1}^q l_{P_q}^{\mathcal{G}_q}$ is a learnable linear function of the logarithmic Pauli fidelities. Notice that $f(\boldsymbol{l})$ exactly corresponds to the circuit of z according to the natural isometry between F and $C_1(G)$. This tells us that every circuit in G indeed corresponds to a learnable linear function. Combining with the fact that the circuits in G span the cycle space $Z(G)$, and the fact that learnable functions are closed under linear combination (Lemma 1), we conclude that $Z(G) \subseteq F_L$.

$F_L \subseteq Z(G)$: For this part, we just need to show that F_L is orthogonal to the cut space $U(G)$, which is the orthogonal complement of the cycle space $Z(G)$. To show this, we will construct a gauge transformation for each element of $U(G)$. The definition of learnability then requires a learnable linear function to be orthogonal to all gauge transformations, thus orthogonal to the entire cut space.

Consider a cut $V = V_1 \cup V_2$ (such that there is at least one edge between V_1 and V_2). We define the *gauge transform map* $\mathcal{M}$ as the following Pauli diagonal map,

$$\mathcal{M}(P) \coloneqq \begin{cases} \eta P, & \text{if } \mathrm{pt}(P) \in V_1, \\ P, & \text{if } \mathrm{pt}(P) \in V_2, \end{cases} \quad \forall P \in \mathsf{P}^n, \tag{31}$$

for a positive parameter $\eta \neq 1$. The gauge transformation induced by $\mathcal{M}$ is defined in the same way as Eq. (10). We will show that there exists two noise models satisfying all the assumptions that are related by a gauge transformation (thus indistinguishable) but yields different values for the function corresponding to the cut $V_1 \cup V_2$.

Starting with a noise model $\mathcal{N} = \{\mathcal{E}^S, \mathcal{E}^M, \Lambda\}$, we first calculate the gauge transformed noise model $\mathcal{N}'$. The SPAM noise channels are transformed as

$$\mathcal{E}^{S'} = \mathcal{M}\mathcal{E}^S, \quad \mathcal{E}^{M'} = \mathcal{E}^M\mathcal{M}^{-1}, \tag{32}$$

which are still Pauli diagonal maps. Using exactly the same argument as in the proof of Theorem 1, by choosing η to be sufficiently close to 1, these transformed maps are guaranteed to be CPTP and satisfy Assumption 4.

Secondly, the single-qubit unitaries are transformed as $\mathcal{U}' = \mathcal{M}\mathcal{U}\mathcal{M}^{-1}$. Calculate the following inner product for any $P, Q \in \mathsf{P}^n$,

$$\begin{aligned} \mathrm{Tr}(P \cdot \mathcal{U}'(Q)) &= \mathrm{Tr}(\mathcal{M}^\dagger(P) \cdot \mathcal{U}(\mathcal{M}^{-1}(Q))) \\ &= \eta^{\mathbf{1}_{V_1}[\mathrm{pt}(P)]}(\eta^{-1})^{\mathbf{1}_{V_1}[\mathrm{pt}(Q)]}\,\mathrm{Tr}(P \cdot \mathcal{U}(Q)). \end{aligned} \tag{33}$$

Here $\mathbf{1}_{V_1}$ is the indicator function of V_1. We see that $\mathrm{Tr}(P \cdot \mathcal{U}'(Q)) = \mathrm{Tr}(P \cdot \mathcal{U}(Q))$ if $\mathrm{pt}(P) = \mathrm{pt}(Q)$. A crucial observation is that a product of single-qubit unitaries can never change the pattern of the input Pauli. More precisely, $\mathcal{U}(Q)$ is a linear combination of Pauli operators with the same pattern as Q. Therefore, if $\mathrm{pt}(P) \neq \mathrm{pt}(Q)$, we would have $\mathrm{Tr}(P \cdot \mathcal{U}'(Q)) = \mathrm{Tr}(P \cdot \mathcal{U}(Q)) = 0$. Combining the two cases, we conclude $\mathcal{U}' = \mathcal{U}$, *i.e.*, the single-qubit unitaries are still noiseless in $\mathcal{N}'$.

Finally, the noisy Clifford gates are transformed as

$$\begin{aligned} \widetilde{\mathcal{G}}' &= \mathcal{M}\mathcal{G}\Lambda_{\mathcal{G}}\mathcal{M}^{-1} \\ &= \mathcal{G}\mathcal{G}^{-1}\mathcal{M}\mathcal{G}\Lambda_{\mathcal{G}}\mathcal{M}^{-1} \\ &=: \mathcal{G}\Lambda'_{\mathcal{G}} \end{aligned} \tag{34}$$

where the transformed noise channel $\Lambda'_{\mathcal{G}} \coloneqq \mathcal{G}^{-1}\mathcal{M}\mathcal{G}\Lambda_{\mathcal{G}}\mathcal{M}^{-1}$ is a Pauli diagonal map. We now calculate its Pauli eigenvalues. For $P \in \mathsf{P}^n$,

$$\begin{aligned} \Lambda'_{\mathcal{G}}(P) &= \mathcal{G}^{-1}\mathcal{M}\mathcal{G}\Lambda_{\mathcal{G}}\mathcal{M}^{-1}(P) \\ &= \eta^{\mathbf{1}_{V_1}[\mathrm{pt}(\mathcal{G}(P))]}(\eta^{-1})^{\mathbf{1}_{V_1}[\mathrm{pt}(P)]}\lambda_P^{\mathcal{G}}\, P \\ &= \begin{cases} \eta\lambda_P^{\mathcal{G}}, & \mathrm{pt}(P) \in V_1,\ \mathrm{pt}(\mathcal{G}(P)) \in V_2. \\ \eta^{-1}\lambda_P^{\mathcal{G}}, & \mathrm{pt}(P) \in V_2,\ \mathrm{pt}(\mathcal{G}(P)) \in V_1. \\ \lambda_P^{\mathcal{G}}, & \text{otherwise}. \end{cases} \end{aligned} \tag{35}$$

Again, Assumption 4 guarantees that $\Lambda'_{\mathcal{G}}$ is a CPTP map satisfying all of our noise assumptions as long as η is sufficiently close to 1. We omit the argument here as it is the same as in the previous proof. Define $t_p \coloneqq \log \eta$ where p denotes the cut $V_1 \cup V_2$. The above gauge transformation of the log Pauli fidelity can be written as

$$\boldsymbol{l}' = \boldsymbol{l} + t_p \boldsymbol{v}_p \tag{36}$$

where $\boldsymbol{v}_p$ is the cut vector of $V = V_1 \cup V_2$ as defined in Eq. (24).

We have just defined a gauge transformation $\mathcal{M}_p$ for an arbitrary cut p. Fix a basis of the cut space B (where vectors in B has the form in Eq. (24)). For a generic element of the cut space $\boldsymbol{v} \in U(G)$, we can decompose it as $\boldsymbol{v} = \sum_{p \in B} t_p \boldsymbol{v}_p$ ($t_p \in \mathbb{R}$). We define the gauge transformation $\mathcal{M}_{\boldsymbol{v}}$ associated with $\boldsymbol{v}$ as a consecutive application of the gauge transformations $\{\mathcal{M}_p\}$ for each $p \in B$, each with parameter t_p. Here we assume that each $|t_p|$ is sufficiently small, as otherwise we can rescale the vector. This implies that $\mathcal{M}_{\boldsymbol{v}}$ is a valid gauge transformation. The effect of such a transformation is

$$\boldsymbol{l}' = \boldsymbol{l} + \boldsymbol{v}. \tag{37}$$

Now, Definition 1 implies that a learnable function $\boldsymbol{f}$ must remain unchanged under gauge transformations (as they result in indistinguishable noise models), which means that $\boldsymbol{f} \cdot \boldsymbol{l}' = \boldsymbol{f} \cdot \boldsymbol{l}$. Thus, for all $\boldsymbol{f} \in F_L$, and all $\boldsymbol{v} \in U(G)$, we must have

$$\boldsymbol{f} \cdot \boldsymbol{v} = \boldsymbol{f} \cdot \boldsymbol{l}' - \boldsymbol{f} \cdot \boldsymbol{l} = 0. \tag{38}$$

That is, F_L must be orthogonal to the cut space $U(G)$. According to Lemma 2, $Z(G)$ is the orthogonal complement of $U(G)$, so we conclude that $F_L \subseteq Z(G)$. This completes the second part of our proof.

□

D. Learnability under no-crosstalk assumption

As we commented before, the way we define the gate noise captures a general form of crosstalk [14]. One may ask, if we further make a favorable assumption that gate noise has no crosstalk, would this make the learning of noise much easier. To consider this rigorously, we introduce the following optional assumption. See Fig. S2 for an illustration.

- **Assumption 5** (No crosstalk.) For any $\mathcal{G} \in \mathfrak{G}$ that acts non-trivially only on a k-qubit subspace, the associated Pauli noise channel also acts non-trivially only on that subspace. In other words, if $\mathcal{G} = \mathcal{G}' \otimes \mathcal{I}$, we have $\widetilde{\mathcal{G}} = (\mathcal{G}' \circ \Lambda_{\mathcal{G}}) \otimes \mathcal{I}$ where $\Lambda_{\mathcal{G}}$ is an k-qubit Pauli channel depending only on $\mathcal{G}$ and the (ordered) subset of qubits on which $\mathcal{G}$ acts.

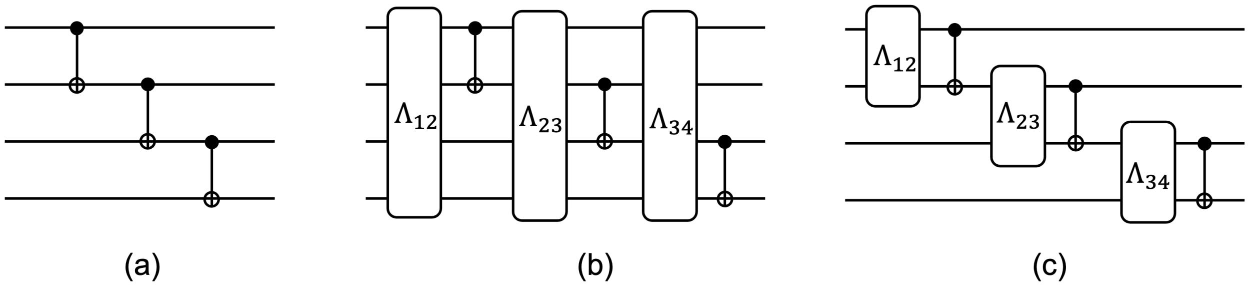

Supplementary Figure S2. Illustration of the crosstalk model. (a) A 4-qubit circuit consists of three ideal CNOT gates. (b) Full crosstalk. The noise channels are 4-qubit and depends on the qubits the CNOT acts on. (c) No crosstalk. The noise channel only acts on a 2-qubit subspace. It can still depend on the qubits the CNOT acts on.

Assumption 5 reduces the number of independent parameters of a noise model. One might expect certain unlearnable functions to become learnable with this assumption. Here, we show that

the simple criteria of learnablity given in Theorem 1 still hold even in this case, as stated in the following proposition.

Proposition 4. *With Assumption 1-5, for any k-qubit Clifford gate $\mathcal{G}$ and Pauli operator P_a, the Pauli fidelity $\lambda_a^{\mathcal{G}}$ is unlearnable if and only if $\mathcal{G}$ changes the pattern of P_a, i.e.,* $\mathrm{pt}(\mathcal{G}(P_a)) \neq \mathrm{pt}(P_a)$.

Proof. We just need to modify the proof of Theorem 1. For the "only if" part, restrict our attention to the k-qubit subsystem that $\mathcal{G}$ acts on, and do a cycle benchmarking protocol as in the original proof. We can easily conclude that $\lambda_a^{\mathcal{G}}$ is learnable if $\mathrm{pt}(P_a) = \mathrm{pt}(\mathcal{G}(P_a))$.

For the "if" part, construct the same gauge transformation map as in the original proof. That is, for an index $i \in [n]$ such that $\mathrm{pt}(P_a)_i \neq \mathrm{pt}(\mathcal{G}(P_a))_i$, let $\mathcal{M} = \mathcal{D}_i \otimes \mathcal{I}_{[n]\setminus i}$ where D_i is the single-qubit deplorizing channel on the ith qubit with some parameter η. With the no-crosstalk assumption, a generic k-qubit noisy Clifford gate $\widetilde{\mathcal{T}}$ transforms as

$$\widetilde{\mathcal{T}} \otimes \mathcal{I} \mapsto \mathcal{M} \circ (\widetilde{\mathcal{T}} \otimes \mathcal{I}) \circ \mathcal{M}^{-1}. \tag{39}$$

If $\mathcal{T}$ does not act on the ith qubit, $\mathcal{M}$ commutes with $\widetilde{\mathcal{T}}$ and the noisy Clifford gate remains unchanged. If $\mathcal{T}$ acts non-trivially on the ith qubit,

$$\widetilde{\mathcal{T}} \otimes \mathcal{I} \mapsto (\mathcal{D}_i \circ \widetilde{\mathcal{T}} \circ \mathcal{D}_i^{-1}) \otimes \mathcal{I}. \tag{40}$$

This means the transformed noise channel acts non-trivially only on the k-qubit subsystem that $\mathcal{G}$ acts on, thus satisfies the no-crosstalk assumption. The Pauli fidelities of the noise channel will be updated as Eq. (15). Following the same argument of the original proof, we conclude that $\lambda_a^{\mathcal{G}}$ is unlearnable if $\mathrm{pt}(P_a) \neq \mathrm{pt}(\mathcal{G}(P_a))$. □

It is also possible to generalize the graph theoretical characterization in Theorem 2 to the no-crosstalk case. One challenge in this case is that, different edges in the pattern transfer graph no longer stand for independent variables. For example, consider a 3-qubit system and a CNOT on the first two qubits. Since $\mathrm{CNOT}(XI) = XX$, we would have the following two edges in the pattern transfer graph

$$e_{XII,\mathrm{CNOT}\otimes\mathcal{I}} = (100, 110), \quad e_{XIX,\mathrm{CNOT}\otimes\mathcal{I}} = (101, 111).$$

However, with the no-crosstalk assumption, we have

$$\lambda_{XII}^{\mathrm{CNOT}\otimes\mathcal{I}} = \lambda_{XIX}^{\mathrm{CNOT}\otimes\mathcal{I}} = \lambda_{XI}^{\mathrm{CNOT}}, \tag{41}$$

which means the above two edges represent the same Pauli fidelity. As a result, a gauge transformation (as defined in the proof of Theorem 2) that changes λ_{XII} and λ_{XIX} differently is no longer a valid transformation. In other word, a cut represents a valid gauge transformation only if it cuts through all the edges for the same Pauli fidelity simultaneously. This could decrease the number of unlearnable degrees of freedom. We leave the precise characterization of the learnable space with no-crosstalk assumptions as an open question. It is also interesting to study the learnability under other practical assumptions about the Pauli noise model, such as the sparse Pauli-Lindbladian model [8] and the Markovian graph model [1, 15].

E. Learnability of Pauli error rates

We have been focusing on the learnability of Pauli fidelities $\boldsymbol{\lambda}$. One may ask similar questions about Pauli error rates $\boldsymbol{p}$. It turns out that, at least in the weak-noise regime (*i.e.*, λ_a close to 1),

the learnability of $\boldsymbol{p}$ is $\boldsymbol{\lambda}$ are highly related. To see this, note that

$$\begin{aligned} p_a &= \frac{1}{4^n}\sum_b (-1)^{\langle a,b\rangle}\lambda_b \\ &\approx \frac{1}{4^n}\sum_b (-1)^{\langle a,b\rangle}(\log\lambda_b + 1) \\ &= \frac{1}{4^n}\sum_b (-1)^{\langle a,b\rangle} l_b + \delta_{a,\mathbf{0}}, \end{aligned} \tag{42}$$

which means that p_a is approximately a linear function of the logarithmic Pauli fidelity $\boldsymbol{l}$. Therefore, one can in principle use Theorem 2 to completely decide the learnability of any Pauli error rates (with weak-noise approximation). Furthermore, since the Walsh-Hadamard transformation is invertible, different p_a corresponds to linearly-independent function of $\boldsymbol{l}$. This means that the number of linearly independent equations we can obtain about the Pauli error rates is the same as the learnable degrees of freedom of the Pauli fidelities. In Table S3, we list a basis for all the learnable Pauli fidelities/Pauli error rates. One can see that there is an exact correspondence between these two. We leave a fully general argument for future study.

Learnable log Pauli fidelities	$l_{II}, l_{ZI}, l_{IX}, l_{ZX}, l_{XZ}, l_{YY}, l_{XY}, l_{YZ}$,<br>$l_{IZ}+l_{ZZ}, l_{IY}+l_{ZY}, l_{IZ}+l_{ZY}, l_{XI}+l_{XX}, l_{YI}+l_{YX}, l_{XI}+l_{YX}$
Learnable Pauli error rates (approximately)	$p_{II}, p_{ZI}, p_{IX}, p_{ZX}, p_{XZ}, p_{YY}, p_{XY}, p_{YZ}$,<br>$p_{IZ}+p_{ZZ}, p_{IY}+p_{ZY}, p_{IZ}+p_{ZY}, p_{XI}+p_{XX}, p_{YI}+p_{YX}, p_{XI}+p_{YX}$

Supplementary Table S3. A complete basis for the learnable linear functions of log Pauli fidelities and Pauli error rates (the latter is approximate) for a single CNOT gate.

S3. ADDITIONAL DETAILS ABOUT THE NUMERICAL SIMULATIONS

In this section, we provide more details about the numerical simulations mentioned in the main text. The simulation is conducted using `qiskit` [16], an open-source Python package for quantum computing. We simulate a two-qubit system where single-qubit Clifford gates are noiseless, and CNOT is subject to amplitude damping channels on both qubits. Note that amplitude damping is not Pauli noise, but we apply randomized compiling and will only estimate its Pauli diagonal part. We also note that, `qiskit` adds the noise channel *after* gate by default, but our theory assume the noise to be *before* gate. These two models can be easily converted between each other via

$$\mathcal{G}\circ\Lambda_{\mathcal{G}} = (\mathcal{G}\circ\Lambda_{\mathcal{G}}\circ\mathcal{G}^\dagger)\circ\mathcal{G} = \Lambda'_{\mathcal{G}}\circ\mathcal{G}. \tag{43}$$

If $\mathcal{G}$ is Clifford, $\Lambda_{\mathcal{G}}$ is a Pauli channel if and only if $\Lambda'_{\mathcal{G}}$ is a Pauli channel. In the following, we will be consistent with our theory and assume the noise to be before gate. Besides, we let the measurement to have 0.3% bit-flip rate on each qubit and the state-preparation to be noiseless.

Fig. S3 shows the estimates collected using standard CB and interleaved CB (circuits shown in Fig. 1 of main text). Compared to the true values, we see that both simulations yields accurate predictions of the learnable Pauli fidelities.

Fig. S4 (a) calculates the physically feasible region according to the estimates in terms of $\{\lambda_{XX}, \lambda_{ZZ}\}$, using approaches discussed in the main text. Due to the special structure of the twirled amplitude damping noise (no Z-error), the feasible region for λ_{XX} is extremely narrow. To eliminate the effect of statistical error, we allow a smoothing parameter ε in calculating the physical region, making the constraints to be $p_a \geq -\varepsilon$. Here ε is chosen to be the largest standard deviation

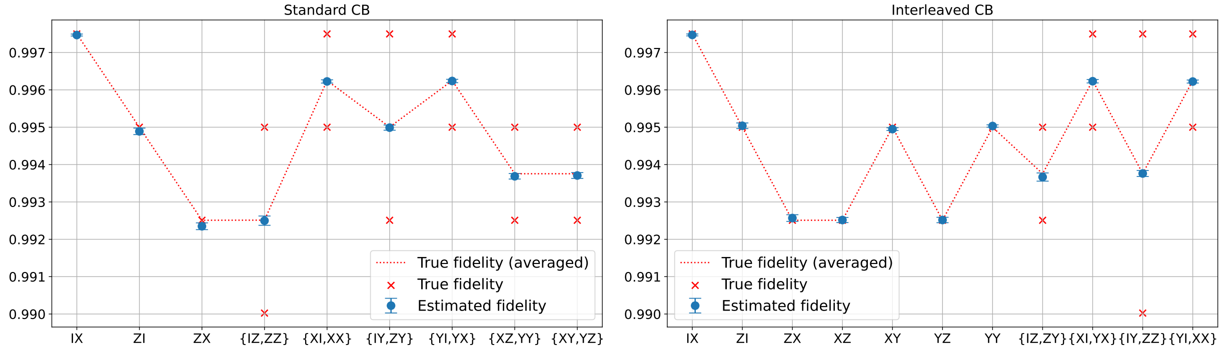

Supplementary Figure S3. Numerical estimates of Pauli fidelities of a CNOT gate via standard CB (left) and CB with interleaved gates (right), using circuits shown in Fig. 1 of main text. Each Pauli fidelity is fitted using seven different circuit depths $L = [2, 2^2, ..., 2^7]$. For each depth $C = 30$ random circuits and 200 shots of measurements are used. The red cross shows the true fidelities and the red dash line shows the average of true fidelities within any two-Pauli group.

in estimating the learnable Pauli fidelities. In Fig. S4 (b)(c) we see that the true fidelity indeed falls into the physical region and is actually close to the lower-left corner of the physical region.

Fig. S5 shows the simulation results of intercept CB. We see that, we obtain an accurate estimate even for the unlearnable Pauli fidelities. Besides, the estimate lies inside the physically feasible region up to a standard deviation. This shows that intercept CB should work well in resolving the unlearnability if we do have access to noiseless state-preparation (and the method is robust against measurement noise). Therefore, failure of this method in experiment implies a non-negligible state-preparation error, as discussed in the main text.

S4. JUSTIFICATION FOR THE CLAIM IN SEC. S2 C

We claim in Sec. S2 C that any measurement probability generated in experiment can be expressed as a polynomial of Pauli fidelities, and that each term in the polynomial can be learned in a CB experiment. This is the motivation why we only care for a single monomial of Pauli fidelities. Here we justify this claim.

Consider the most general experimental design: prepare some initial state ρ_0, apply some quantum circuit $\mathcal{C}$, and conduct a POVM measurement $\{E_j\}_j$. Denote the noisy realization of these objects with a tilde. Because of noise, the probability of obtaining a certain measurement outcome j is

$$\Pr(j) = \mathrm{Tr}\left(\widetilde{E}_j\widetilde{\mathcal{C}}(\widetilde{\rho}_0)\right) = \mathrm{Tr}\left(E_j\left(\Lambda^M\circ\widetilde{\mathcal{C}}\circ\Lambda^S\right)(\rho_0)\right) \equiv \mathrm{Tr}\left(E_j\rho'\right). \quad (44)$$

Here Λ^S, Λ^M are the noise channels for state preparation and measurement, respectively. The Pauli fidelity of them are denoted by λ_a^S, λ_a^M for Pauli operator a, respectively. We define $\rho' := (\Lambda^M\circ\widetilde{\mathcal{C}}\circ\Lambda^S)(\rho_0)$ which encodes all the information that can be extracted from a quantum measurements. We will obtain a general formula for ρ'.

First note that a general noisy quantum circuit $\widetilde{\mathcal{C}}$ satisfying our assumptions can be expressed as

$$\widetilde{\mathcal{C}} = C^{(m)}\circ\widetilde{\mathcal{G}}_m\circ\cdots\circ C^{(1)}\circ\widetilde{\mathcal{G}}_1\circ C^{(0)}, \quad (45)$$

where $\mathcal{G}_j \in \mathfrak{G}$ is an n-qubit Clifford gate and $C^{(j)}$ is the tensor product of single-qubit gates. A crucial property for single-qubit gates is that they never change the Pauli pattern. More rigorously,

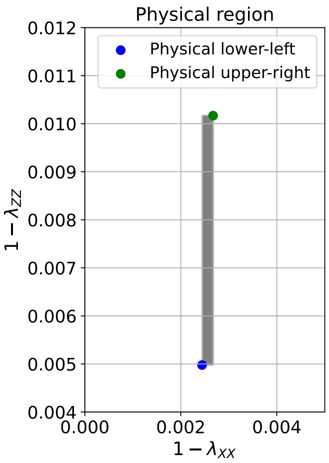

(a) feasible region

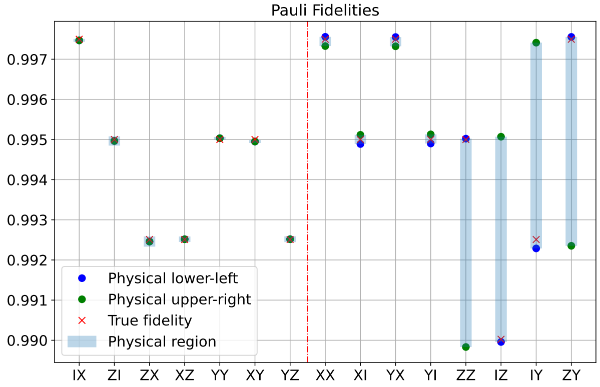

(b) Pauli fidelities

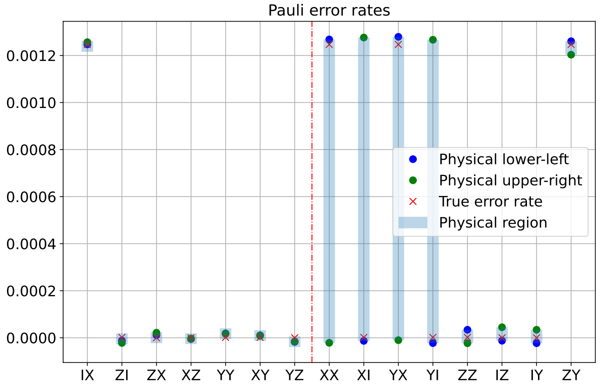

(c) Pauli errors

Supplementary Figure S4. Feasible region of the learned Pauli noise model, using data from Fig. S3. (a) Feasible region of the unlearnable degrees of freedom in terms of λ_{XX} and λ_{ZZ}. (b) Feasible region of individual Pauli fidelities. (c) Feasible region of individual Pauli errors.

one have that

$$C^{(j)}(P_a) = \sum_{b\sim \mathrm{pt}(a)} c^{(j)}_{b,a} P_b, \quad \forall P_a \in \mathsf{P}^n, \tag{46}$$

where $c^{(j)}_{b,a} \in \mathbb{R}$, and the summation is over all P_b that have the same Pauli pattern as P_a.

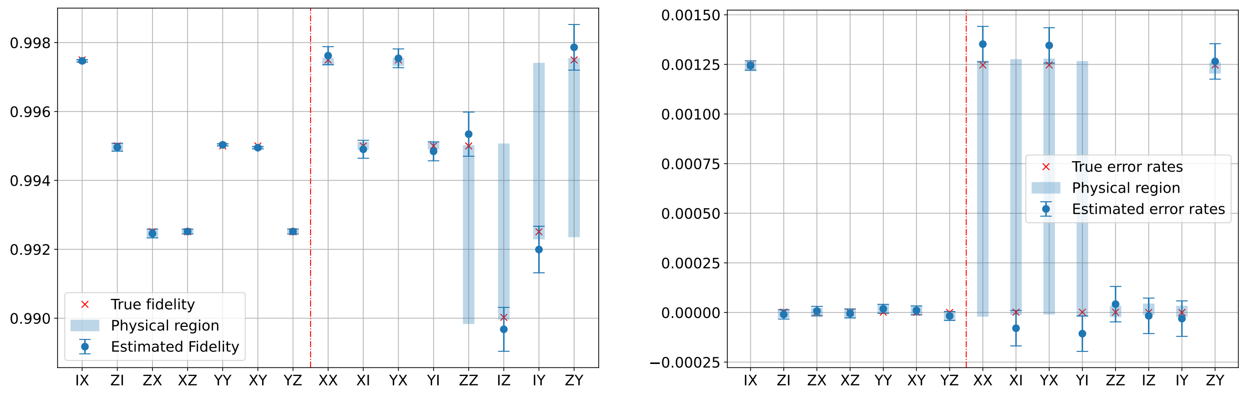

(a) (b)

Supplementary Figure S5. The learned Pauli noise model using intercept CB. The feasible region (blue bars) are taken from Fig. S4. Estimates of Pauli fidelities (a) and Pauli error rates (b). Each data point is fitted using seven different circuit depths $L = [2, 2^2, ..., 2^7]$. For each depth $C = 300$ random circuits and 2000 shots of measurements are used.

Now consider the action of $\widetilde{\mathcal{C}}$ on an arbitrary Pauli operator P_a.

$$
\begin{aligned}
\widetilde{\mathcal{C}}(P_a) &= (C^{(m)} \circ \widetilde{\mathcal{G}}_m \circ \cdots \circ C^{(1)} \circ \widetilde{\mathcal{G}}_1 \circ C^{(0)})(P_a) \\
&= (C^{(m)} \circ \widetilde{\mathcal{G}}_m \circ \cdots \circ C^{(1)} \circ \widetilde{\mathcal{G}}_1)\left(\sum_{b_0 \sim \mathrm{pt}(a)} c^{(0)}_{b_0,a} P_{b_0}\right) \\
&= (C^{(m)} \circ \widetilde{\mathcal{G}}_m \circ \cdots \circ C^{(1)})\left(\sum_{b_0 \sim \mathrm{pt}(a)} c^{(0)}_{b_0,a} \lambda^{\mathcal{G}_1}_{b_0} P_{\mathcal{G}_1(b_0)}\right) \\
&= (C^{(m)} \circ \widetilde{\mathcal{G}}_m \circ \cdots \circ C^{(2)})\left(\sum_{\substack{b_0 \sim \mathrm{pt}(a), \\ b_1 \sim \mathrm{pt}(\mathcal{G}_1(b_0))}} c^{(1)}_{b_1,\mathcal{G}_1(b_0)} c^{(0)}_{b_0,a} \lambda^{\mathcal{G}_2}_{b_1} \lambda^{\mathcal{G}_1}_{b_0} P_{\mathcal{G}_2(b_1)}\right) \\
&= \cdots \\
&= \sum_{\substack{b_0 \sim \mathrm{pt}(a), \\ b_1 \sim \mathrm{pt}(\mathcal{G}_1(b_0)), \\ \cdots \\ b_m \sim \mathrm{pt}(\mathcal{G}_m(b_{m-1}))}} c^{(m)}_{b_m,\mathcal{G}_m(b_{m-1})} \cdots c^{(1)}_{b_1,\mathcal{G}_1(b_0)} c^{(0)}_{b_0,a} \lambda^{\mathcal{G}_m}_{b_{m-1}} \cdots \lambda^{\mathcal{G}_2}_{b_1} \lambda^{\mathcal{G}_1}_{b_0} P_{b_m}.
\end{aligned}
\tag{47}
$$

For any initial state ρ_0, we can decompose it via Pauli operators as

$$
\rho_0 = \frac{1}{2^n} I + \sum_{a \neq \mathbf{0}} \alpha_a P_a. \tag{48}
$$

Going through the state preparation noise, the quantum circuit, and the measurement noise, the

state evolves to

$$
\begin{aligned}
\rho' &= (\Lambda^M \circ \widetilde{\mathcal{C}} \circ \Lambda^S)(\frac{1}{2^n} I + \sum_{a\neq \mathbf{0}} \alpha_a P_a) \\
&= \frac{1}{2^n} I + \sum_{a\neq \mathbf{0}} \alpha_a \sum_{\substack{b_0\sim \mathrm{pt}(a),\\ b_1\sim \mathrm{pt}(\mathcal{G}_1(b_0)),\\ \cdots\\ b_m\sim \mathrm{pt}(\mathcal{G}_m(b_{m-1}))}} c^{(m)}_{b_m,\mathcal{G}_m(b_{m-1})}\cdots c^{(1)}_{b_1,\mathcal{G}_1(b_0)} c^{(0)}_{b_0,a}\ \lambda^M_{\mathrm{pt}(b_m)}\lambda^{\mathcal{G}_m}_{b_{m-1}}\cdots\lambda^{\mathcal{G}_2}_{b_1}\lambda^{\mathcal{G}_1}_{b_0}\lambda^S_{\mathrm{pt}(a)} P_{b_m} \\
&\equiv \frac{1}{2^n} I + \sum_{a\neq \mathbf{0}} \alpha_a \sum_{\substack{b_0\sim \mathrm{pt}(a),\\ b_1\sim \mathrm{pt}(\mathcal{G}_1(b_0)),\\ \cdots\\ b_m\sim \mathrm{pt}(\mathcal{G}_m(b_{m-1}))}} c^{(m)}_{b_m,\mathcal{G}_m(b_{m-1})}\cdots c^{(1)}_{b_1,\mathcal{G}_1(b_0)} c^{(0)}_{b_0,a}\ \Gamma_{\boldsymbol{b},a} P_{b_m}.
\end{aligned} \tag{49}
$$

Here we define $\Gamma_{\boldsymbol{b},a} = \lambda^M_{\mathrm{pt}(b_m)}\lambda^{\mathcal{G}_m}_{b_{m-1}}\cdots\lambda^{\mathcal{G}_2}_{b_1}\lambda^{\mathcal{G}_1}_{b_0}\lambda^S_{\mathrm{pt}(a)}$, which is a monomial of Pauli fidelities. The measurement outcome probability $\Pr(j)$ is a linear combination of such $\Gamma_{\boldsymbol{b},a}$ plus some constant. Moreover, each $\Gamma_{\boldsymbol{b},a}$ of the above form can also be learned from a simple experiment, by choosing the initial state to be a $+1$ eigenstate of P_a, measurement operator to be P_{b_m}, and $C^{(j)}$ to be the product of single-qubit Clifford gates satisfying $C^{(j)}(\mathcal{G}_j(b_{j-1})) = b_j$ (which is possible because $\mathrm{pt}(b_j) = \mathrm{pt}(\mathcal{G}_j(b_{j-1})))$. Therefore, to completely characterize a noise model, we only need to extract the products of Pauli fidelities in the form of $\Gamma_{\boldsymbol{b},a}$. This justifies our earlier claim.

SUPPLEMENTARY REFERENCES

[1] S. T. Flammia and J. J. Wallman, Efficient estimation of pauli channels, ACM Transactions on Quantum Computing **1**, 10.1145/3408039 (2020).

[2] S. T. Flammia and R. O'Donnell, Pauli error estimation via population recovery, Quantum **5**, 549 (2021).

[3] S. Chen, W. Yu, P. Zeng, and S. T. Flammia, Robust shadow estimation, PRX Quantum **2**, 030348 (2021).

[4] E. Nielsen, J. K. Gamble, K. Rudinger, T. Scholten, K. Young, and R. Blume-Kohout, Gate Set Tomography, Quantum **5**, 557 (2021).

[5] A. Erhard, J. J. Wallman, L. Postler, M. Meth, R. Stricker, E. A. Martinez, P. Schindler, T. Monz, J. Emerson, and R. Blatt, Characterizing large-scale quantum computers via cycle benchmarking, Nature Communications **10**, 5347 (2019).

[6] J. J. Wallman and J. Emerson, Noise tailoring for scalable quantum computation via randomized compiling, Physical Review A **94**, 052325 (2016).

[7] A. Hashim, R. K. Naik, A. Morvan, J.-L. Ville, B. Mitchell, J. M. Kreikebaum, M. Davis, E. Smith, C. Iancu, K. P. O'Brien, I. Hincks, J. J. Wallman, J. Emerson, and I. Siddiqi, Randomized compiling for scalable quantum computing on a noisy superconducting quantum processor, Phys. Rev. X **11**, 041039 (2021).

[8] E. v. d. Berg, Z. K. Minev, A. Kandala, and K. Temme, Probabilistic error cancellation with sparse pauli-lindblad models on noisy quantum processors (2022), arXiv:2201.09866 [quant-ph].

[9] S. Ferracin, A. Hashim, J.-L. Ville, R. Naik, A. Carignan-Dugas, H. Qassim, A. Morvan, D. I. Santiago, I. Siddiqi, and J. J. Wallman, Efficiently improving the performance of noisy quantum computers (2022), arXiv:2201.10672 [quant-ph].

[10] E. Nielsen, K. Young, and R. Blume-Kohout, First-order gauge-invariant error rates in quantum processors, Bulletin of the American Physical Society (2022).

[11] S. T. Flammia, Averaged circuit eigenvalue sampling (2021), arXiv:2108.05803 [quant-ph].

[12] P. M. Gleiss, J. Leydold, and P. F. Stadler, Circuit bases of strongly connected digraphs, Discuss. Math. Graph Theory **23**, 241 (2003).

[13] B. Bollobás, *Modern graph theory*, Vol. 184 (Springer Science & Business Media, 1998).

[14] M. Sarovar, T. Proctor, K. Rudinger, K. Young, E. Nielsen, and R. Blume-Kohout, Detecting crosstalk errors in quantum information processors, Quantum **4**, 321 (2020).

[15] R. Harper, S. T. Flammia, and J. J. Wallman, Efficient learning of quantum noise, Nature Physics **16**, 1184 (2020).

[16] M. S. A. et al., Qiskit: An open-source framework for quantum computing (2021).
